# Sweet Scents: Nectar Specialist Yeasts Enhance Nectar Attraction of a Generalist Aphid Parasitoid Without Affecting Survival

**DOI:** 10.3389/fpls.2018.01009

**Published:** 2018-07-16

**Authors:** Islam S. Sobhy, Dieter Baets, Tim Goelen, Beatriz Herrera-Malaver, Lien Bosmans, Wim Van den Ende, Kevin J. Verstrepen, Felix Wäckers, Hans Jacquemyn, Bart Lievens

**Affiliations:** ^1^Laboratory for Process Microbial Ecology and Bioinspirational Management, Department of Microbial and Molecular Systems, KU Leuven, Sint-Katelijne Waver, Belgium; ^2^Department of Plant Protection, Faculty of Agriculture, Suez Canal University, Ismailia, Egypt; ^3^VIB Lab for Systems Biology and Centre of Microbial and Plant Genetics Lab for Genetics and Genomics, Department of Microbial and Molecular Systems, KU Leuven, Leuven, Belgium; ^4^Laboratory of Molecular Plant Biology, Biology Department, KU Leuven, Leuven, Belgium; ^5^Biobest, Westerlo, Belgium; ^6^Lancaster Environment Centre, Lancaster University, Lancaster, United Kingdom; ^7^Laboratory of Plant Conversation and Population Biology, Biology Department, KU Leuven, Leuven, Belgium

**Keywords:** floral nectar, *Metschnikowia*, nectar chemistry, microbial volatile (MVOC), *Aphidius ervi*, behavioral response, nectar intake, survival

## Abstract

Floral nectar is commonly inhabited by microorganisms, mostly yeasts and bacteria, which can have a strong impact on nectar chemistry and scent. Yet, little is known about the effects of nectar microbes on the behavior and survival of insects belonging to the third trophic level such as parasitoids. Here, we used five nectar-inhabiting yeast species to test the hypothesis that yeast species that almost solely occur in nectar, and therefore substantially rely on floral visitors for dispersal, produce volatile compounds that enhance insect attraction without compromising insect life history parameters, such as survival. Experiments were performed using two nectar specialist yeasts (*Metschnikowia gruessii* and *M. reukaufii*) and three generalist species (*Aureobasidium pullulans, Hanseniaspora uvarum*, and *Sporobolomyces roseus*). *Saccharomyces cerevisiae* was included as a reference yeast. We compared olfactory responses of the generalist aphid parasitoid *Aphidius ervi* (Haliday) (Hymenoptera: Braconidae) when exposed to these microorganisms inoculated in synthetic nectar. Nectar-inhabiting yeasts had a significant impact on nectar chemistry and produced distinct volatile blends, some of which were attractive, while others were neutral or repellent. Among the different yeast species tested, the nectar specialists *M. gruessii* and *M. reukaufii* were the only species that produced a highly attractive nectar to parasitoid females, which simultaneously had no adverse effects on longevity and survival of adults. By contrast, parasitoids that fed on nectars fermented with the reference strain, *A. pullulans, H. uvarum or S. roseus* showed shortest longevity and lowest survival. Additionally, nectars fermented by *A. pullulans* or *S. roseus* were consumed significantly less, suggesting a lack of important nutrients or undesirable changes in the nectar chemical profiles. Altogether our results indicate that nectar-inhabiting yeasts play an important, but so far largely overlooked, role in plant-insect interactions by modulating the chemical composition of nectar, and may have important ecological consequences for plant pollination and biological control of herbivorous insects.

## Introduction

As a source of sugars, floral nectar is commonly colonized by nectarivorous microbes, most often yeasts and bacteria that may rapidly reach high densities within floral nectar (Herrera et al., 2009; Lievens et al., 2015; Pozo et al., 2015b). Although their precise ecological role is not yet entirely clear (Herrera, 2017), nectar-inhabiting microorganisms have a strong effect on nectar chemistry by altering the concentration and composition of sugars and amino acids (Herrera et al., 2008; de Vega et al., 2009; Canto and Herrera, 2012; Peay et al., 2012; Vannette et al., 2013; Lenaerts et al., 2017), and influencing acidity (Vannette et al., 2013; Good et al., 2014; Lenaerts et al., 2017). These changes may, in turn, affect the nectar's overall nutritional value and appeal to flower-visiting insects (Schaeffer et al., 2014, 2017; Lenaerts et al., 2017). The metabolic activity of nectar-inhabiting microbes has also been shown to affect other floral traits (Vannette and Fukami, 2016, 2018). Herrera and Pozo (2010), for example, showed that experimental addition of *Metschnikowia* yeasts to the nectar of a winter-blooming plant species (*Helleborus foetidus*) significantly increased the nectar temperature. Warmer nectar could offer energetic advantages for insect thermoregulation, as well as being easier to drink owing to its lower viscosity (Nicolson et al., 2013). Recent evidence also points to nectar-inhabiting microorganisms contributing to nectar scents by the production of volatile compounds (Golonka et al., 2014; Rering et al., 2017). It is generally believed that these microbial volatiles can act as semiochemicals that signal a suitable food source (nectar) or habitat to nectar feeding insects (Wright and Schiestl, 2009; Davis et al., 2013), while the microbes benefit from the insects as vectors for dispersal to new environments (Christiaens et al., 2014). The plants may benefit from the presence of microorganisms through increased insect visitation rates or longer foraging time (Schaeffer et al., 2017), ultimately leading to enhanced plant fitness (Schaeffer and Irwin, 2014). However, effects on plant fitness seem to vary depending on the component of plant reproduction considered (Herrera et al., 2013).

For microbes that strongly rely on animal vectors, such as insects, production of insect-attractive volatiles may be an efficient strategy to rapidly disperse and colonize new habitats, while this would be less needed for generalist microbes that live in a wider variety of habitats and are less reliant on insect vectors (Dzialo et al., 2017). Indeed, it was recently shown that *Metschnikowia reukaufii*, a yeast species that is specialized to thrive in the harsh nectar environment (Lievens et al., 2015; Pozo et al., 2015a) and is largely dependent on flower-visiting insects for dispersal (Belisle et al., 2012; Vannette and Fukami, 2017), produces distinctive volatile compounds. It was also demonstrated that this yeast species is more attractive to honey bees (*Apis mellifera*) than generalist microorganisms (Rering et al., 2017). Nevertheless, as the authors only examined responses of one floral visitor, additional research is needed to generalize these results. Another important group of flower-visiting insects are hymenopteran parasitoids. Parasitic Hymenoptera represent a key factor in regulating natural insect populations, and form an important component in biocontrol programs of insect pests (Narendran, 2001). Like bees, in their adult stage, parasitoid wasps feed on carbohydrate-rich food such as floral nectar to cover their energetic and nutritional needs (Jervis et al., 1993). This makes them ideal candidates for further study of the role of nectar microbes in the foraging behavior of flower-visiting insects. Moreover, social Hymenoptera, such as honey bees and bumble bees, have the disadvantage that isolating them from their social interactions within the colony may negatively impact foraging behavior (Garibaldi et al., 2011), food consumption (Arnold, 1979), and survival (Sitbon, 1967). Furthermore, it can be hypothesized that microorganisms that substantially rely on insect vectors for dispersal should not impair life history parameters of their vectors, such as survival. In a recent study, it was found that different nectar bacteria may have a clear effect on the longevity of flower-visiting insects by altering nectar chemistry (Lenaerts et al., 2017), but it remains unclear whether effects can be related to the ecology of the microorganisms.

Here, we tested the hypothesis that yeast species that almost exclusively occur in nectar and therefore largely depend on floral visitors for dispersal produce volatile compounds that enhance insect attraction, and simultaneously yield a nectar chemistry that does not harm the survival of attracted insects. To this end, we used the nectar specialists *Metschnikowia gruessii* and *M. reukaufii* as model yeasts. In addition, we tested more generalist yeast species such as *Aureobasidium pullulans, Hanseniaspora uvarum* and *Sporobolomyces roseus* (Lievens et al., 2015; Pozo et al., 2015b). *Saccharomyces cerevisiae* (Y182), which is not found in nectar, but is known for its high aroma production and attraction of *Drosophila* flies (Christiaens et al., 2014), was used as a reference. All experiments were performed using the solitary hymenopteran parasitoid *Aphidius ervi* (Haliday), which is a generalist parasitoid of aphids. It feeds preferentially on nectar as a main source of sugars over honeydew (Vollhardt et al., 2010), and its efficiency in suppressing aphid populations is drastically increased by the provision of floral nectar (Araj et al., 2008, 2011). First, we investigated the effect of the different nectar-inhabiting yeasts (NIYs) on the volatile production and chemical composition of a synthetic nectar solution mimicking real nectar. Next, using a binary olfactory choice assay, the parasitoid response to the NIY-fermented nectars was assessed. Finally, using a capillary feeder (CAFE) assay, the intake of NIY-fermented nectars by parasitoid adults and the subsequent impact on their longevity and survival were studied.

## Materials and methods

### Study organisms

#### Yeasts

Five yeast strains that were previously isolated from floral nectar of wild plants were used in this study (Table S1), including two nectar specialist species (*M. gruessii* and *M. reukaufii*) and three generalist species (*A. pullulans, H. uvarum*, and *S. roseus*) (Jacquemyn et al., 2013; Lenaerts et al., 2016b). *Metschnikowia reukaufii* and *M. gruessii* are common and abundant inhabitants of floral nectar that have specialized on the nectar environment (Herrera et al., 2009; Lievens et al., 2015; Pozo et al., 2016). Additionally, both species are strongly dependent on floral visitors for transmission among flowers, including pollinators and parasitoids (e.g., Belisle et al., 2012; Pozo et al., 2012; Herrera et al., 2014; Srinatha et al., 2015). By contrast, the other three yeast species have a broader habitat range and are less dependent on insect vectors for dispersal. More specifically, *A. pullulans* is a ubiquitous yeast-like fungus that can be found in different environments including soil, water, air, and in or on plants (Andrews et al., 1994). *Hanseniaspora uvarum* is an apiculate yeast species that is frequently found on mature fruits (Jolly et al., 2014), whereas *S. roseus* is mostly associated with the phyllosphere (Nakase, 2000). Additionally, as a reference we included a *S. cerevisiae* strain (Y182) that produces strong aroma (e.g., aroma-active esters) and has been shown to attract *Drosophila melanogaster* fruit flies (Christiaens et al., 2014). Yeast strains were stored at −80°C in yeast extract peptone dextrose broth (YPDB; Difco, Le Pont-de-Claix, France) containing 37.5% glycerol.

#### Insects

Experiments were performed with adults of *A. ervi* (Hymenoptera: Braconidae). *Aphidius ervi* is a solitary generalist endoparasitoid that attacks many aphid species, including numerous species of economic importance (van Lenteren, 2012). For all experiments, *A. ervi* mummies were supplied by Biobest (Ervi-system®, Westerlo, Belgium). Upon receiving, mummies were either kept at 4°C for a maximum of 48 h until usage or placed directly in a nylon insect cage (20 × 20 × 20 cm, BugDorm-41515, MegaView Science Co., Ltd., Taichung, Taiwan) and kept under controlled conditions (22°C, 70% relative humidity, 16:8 h light:dark photoperiod) until adult emergence. Prior to starting experiments, insects were subjected to a dark period of 8 h. All experiments were performed with feeding-inexperienced and water-starved adults that were <24 h old.

### Inoculation and fermentation of synthetic nectar

In order to prepare different yeast-fermented nectars, stock cultures were plated on yeast extract peptone dextrose agar (YPDA), followed by a re-streak on the same medium and subsequent incubation for 2 days at 25°C. Yeast strains were thereafter inoculated in a test tube containing 5 ml YPDB and incubated at 25°C on a rotary shaker at 150 rpm. After overnight incubation, cells were washed two times and suspended in sterile physiological water (0.9% NaCl) until an optical density (OD 600 nm) of 1 was reached. Afterwards, 1.5 ml of this suspension was used to inoculate a 250 ml Erlenmeyer flask containing 150 ml sterile synthetic nectar prepared by filter-sterilizing 15% w/v sucrose solution supplemented with 3.16 mM amino acids from digested casein (Vannette and Fukami, 2014; Lenaerts et al., 2017). Erlenmeyer flasks were sealed with fermentation water locks and incubated statically at 25°C for 7 days. The incubation period was determined by regularly monitoring yeast growth to obtain densities that were comparable with those observed in floral nectar (de Vega et al., 2009). Each fermentation was performed in duplicate, and a medium without yeast inoculation was included as a mock control (which was also confirmed to be free of yeasts and bacteria after the fermentation). Following the fermentations, yeast-fermented nectars were centrifuged at 6,000 rpm for 3 min and subsequently filtered (pore size 0.22 μm; Nalgene, Waltham, MA, USA) to obtain cell-free cultures. Cell-free nectar media were then stored in small aliquots in sterile dark glass vials (Fagron, Nazareth, Belgium) at −20°C until further use.

### Impact of yeasts on scent profiles

In order to investigate the effects of the different yeast strains on nectar scent, fermented nectars were subjected to a headspace gas chromatography (GC) analysis coupled with a flame ionization detector (HS-GC-FID; Shimadzu, Kyoto, Japan) as described previously (Christiaens et al., 2014). For each biological replicate of fermented nectars, the analysis was performed with two technical replicates. The GC was calibrated for 15 important yeast specific volatiles, including esters (ethyl acetate, isobutyl acetate, propyl acetate, isoamyl acetate, phenyl ethyl acetate, ethyl propionate, ethyl butyrate, ethyl hexanoate, ethyl octanoate, ethyl decanoate), higher alcohols (isoamyl alcohol, isobutanol, butanol, phenyl ethanol) and acetaldehyde as described in Gallone et al. (2016). The GC was fitted with the DB-WAX column (30 m length × 0.32 mm inner diameter × 0.5 μm film thickness, Agilent Technologies, Santa Clara, CA, USA). Samples of 5 ml fermented nectar were collected in 15 ml glass tubes containing 1.75 g of sodium chloride each. These tubes were immediately closed and cooled to minimize evaporation of volatile compounds. The injector port of the GC instrument was held at 250°C *via* a headspace auto sampler (PAL system; CTC Analytics, Zwingen, Switzerland). N_2_ was used as the carrier gas. The GC oven temperature was programmed at 50°C for 5 min, after which it was increased to 80°C at 5°C min^−1^. Next, the temperature was increased to 200°C at 4°C min^−1^ and held at 200°C for 3 min followed by a final ramp of 4°C min^−1^ till 230°C. Results were analyzed with the GCSolution software version 2.4 (Shimadzu, Kyoto, Japan).

### Impact of yeasts on nectar chemistry

To investigate the effects of the different yeast strains on nectar chemistry, prepared synthetic nectar media were subjected to chemical analyses. In particular, concentrations of sugars and amino acids were determined with a high-performance anion-exchange chromatography with pulsed amperometric detection (HPAEC-PAD; Thermo Fisher Scientific Dionex, Sunnyvale, CA, USA) as described by Lenaerts et al. (2016a). Furthermore, the pH was determined with a pH electrode (WTW Inolab, Weilheim, Germany) and the Gallery™ Plus Beermaster (ThermoFisher, Vantaa, Finland) was used to quantify acetic acid, D-Lactic acid and sulfur dioxide levels. Concentrations were calculated from a calibration curve generated using standards according to the manufacturer's instructions. Analyses were performed with two technical replicates per nectar medium (i.e., per biological replicate).

### Impact of yeast-fermented nectars on insect behavioral response

In order to assess the effect of yeast-fermented nectar on *A. ervi* foraging behavior, a behavioral bioassay using a glass Y-tube olfactometer was performed. The olfactometer consisted of a 20-cm-long stem tube with 1.5 cm inner diameter and two 12-cm-long lateral arms with a 60° angle at the Y-junction. Charcoal filtered humidified and purified air was provided at 400 ml min^−1^ (Brooks Instrument flow meter, Hatfield, USA) to both branches of the Y-tube *via* two odor chambers using a vacuum pump (Tetratec APS 150, Mella, Germany). All connections in the olfactometer were made using Teflon tubing (Figure S1). To test a given yeast strain, a filter paper (Macherey-Nagel, Düren, Germany) was loaded with 150 μl cell-free fermented nectar and placed into one of the two odor chambers of the olfactometer, whereas in the second chamber another filter paper was placed on which 150 μl non-inoculated medium was added. The bioassay was carried out by releasing 20 groups of five adult females (*n* = 100), in one experimental day, at the base of the olfactometer and evaluating their response 10 min after their release. Wasps that passed a set line at the end of the olfactometer arms (1 cm from the joint) and remained there at the time of evaluation were considered to have chosen for the odor source connected to that olfactometer arm. Parasitoids that did not make a choice within 10 min after release were considered as non-responding individuals, and were excluded from the statistical analysis. In order to avoid light-bias, the experiment was conducted in a 60 × 40 × 25-cm white chamber that was illuminated with four warm white led 5.5 W lamps (EGLO E27, light intensity 1880 lumens). Further, to avoid positional bias, the odor chambers were rotated after 10 releases with a new set of Teflon tubes. The glass Y-tube was renewed by a cleaned tube (see below) after every five runs, to eliminate choices which may be based on potential insect traces. Filter papers were replaced with fresh filter papers with 150 μl of the tested medium every two runs to maintain a high level of odor release. At the end of the experiment, all olfactometer parts (glass and Teflon tubes) were thoroughly cleaned with tap water and then distilled water, acetone (Forever, Courcelles, Belgium; purity > 99%) and finally pentane (Sigma-Aldrich, Steinheim, Germany; purity 98%). After solvents had evaporated, the glass parts were placed overnight in an oven at 150°C. All bioassays were conducted at 20 ± 1°C, 60 ± 5% RH and performed between 09:00 and 16:00 h.

### Impact of yeast-fermented nectars on nectar intake, longevity, and survival

The capillary feeder (CAFE) assay that was previously developed by Lenaerts et al. (2016a) was used to evaluate the effect of yeast inoculation on nectar intake and parasitoid longevity and survival. In brief, a cylindrical plastic container (height: 12.5 cm; diameter: 10 cm) was provided with four calibrated glass micropipettes (5.0 μl, Blaubrand IntraMARK, Wertheim, Germany) that were filled with 4.0 μl of the cell-free nectar solution fermented by one of the tested yeast strains (no-choice; all four capillaries contained the same nectar solution) and covered with 1.0 μl inert mineral oil to minimize evaporation. Additionally, a treatment with nectar that had not been inoculated with yeast was included as a control. Filled capillaries were inserted through the lid *via* truncated 200-μl yellow pipette tips to orientate the wasps to the microcapillaries (Battaglia et al., 2000). Further, to allow ventilation the lid of the container was pierced and covered with a fine mesh (2.5 × 2.5 cm; mesh size 0.27 × 0.88 mm). To provide parasitoids with sufficient water and humidity, a filter paper saturated with 500 μl of sterile water was put at the container's bottom, which was supplemented daily with an additional 200 μl of sterile water. Experiments were performed using a group of 75 individuals (both males and females) that were divided equally over five containers per treatment (15 individuals per cage) in a controlled environment (Micro Clima-Series™, Economic Lux Chamber, Snijders LABS, Tilburg, The Netherlands). Experiments were conducted at 22°C, 70% RH and a 16:8-h light: dark photoperiod with a light intensity of 100 μmol/m^2^ s during periods of light. For the first 9 h of the experiment, nectar intake was assessed every hour by measuring the nectar column in the microcapillaries using a digital caliper (Mitutoyo Digimatic, resolution 0.01 mm). For each CAFE feeder, consumption values for the four capillaries were summed and subsequently averaged over the five replicates (*n* = 5). To determine the exact starting volume, we also measured the level of nectar solution right before the start of the experiment. An additional identical feeding unit, but without parasitoids, was included for each treatment to establish losses through evaporation. These values were then subtracted from experimental readings to account for evaporative losses.

Further, the effect of the different yeast-fermented nectars on insect longevity (days from adult emergence until death) and survival (number or proportion of adults surviving under the testing conditions) was assessed using the same individuals as those used in the previous analysis (*n* = 75). More specifically, parasitoid longevity was recorded daily by counting and removing the dead individuals in each CAFE container, until the last individual had died. To avoid microbial contamination of the nectar solutions, capillaries were replaced daily.

### Data analysis and visualization

All analyses of nectar volatiles and nectar chemistry were performed using two biological replicates which were analyzed each in duplicate (two technical replicates). Variation between both biological and technical replicates was low (Data Sheet 1), illustrating the robustness of our data, as has also been shown previously (Christiaens et al., 2014). For each biological replicate, we used the mean values of the two technical replicates to run the statistical analysis. First, changes in nectar chemistry (MVOCs, amino acids, sugars and acidity) by yeast fermentation were visualized using a principal component analysis (PCA), incorporating each compound as a variable according to Rencher (2002). We used two types of output: a matrix of “scores,” which provides the location of each sample on each PC, and a matrix of “loadings” which indicates the strength of correlation between individual compounds and each PC. Prior to analysis, data were normalized by sum, cube root transformed and mean-centered, and divided by the standard deviation of each variable before PCA, using the comprehensive online tool suite MetaboAnalyst 3.0 (Xia et al., 2015). Next, to get better insight into the changes in nectar profiles (i.e., MVOCs, amino acids, sugars and acidity) upon yeast inoculation, data were analyzed using one-way ANOVA for each individual compound. Data were first checked for normal distribution and homogeneity of variance by Shapiro–Wilk and by Levene's test, respectively. The obtained *P*-values were adjusted for multiple testing, by the Benjamini and Hochberg (BH) step-up procedure to control the false discovery rate (FDR) (Benjamini and Hochberg, 1995). As differences in nectar chemistry between biological replicates were small, insect experiments were performed using sampled nectar from one of both biological replicates.

To examine the effect of the different nectars on parasitoid foraging behavior, parasitoid response was analyzed under the null hypothesis that adult parasitoids show no preference for either olfactometer arm (i.e., 50:50 response). Data (*n* = 100) were checked first for normality using Shapiro–Wilk test, after which they were analyzed with a *t*-test. The data (*n* = 5) of total nectar consumption during the first 9 h were analyzed using one-way ANOVA and means were then compared using a Student-Newman-Keuls *post-hoc* test. Longevity data (*n* = 75) were analyzed using one-way ANOVA which was followed by a Student-Newman-Keuls *post-hoc* test to compare means. Kaplan-Meier survival analysis and log-rank tests with Bonferroni correction were used to compare the survival of *A. ervi* adults fed on the various yeast-fermented nectars.

All the univariate analyses were performed using the statistical package SigmaPlot 12.3 (SYSTAT Inc., Chicago, IL, USA).

### Ethical note

Experimental manipulation of parasitoids occurred according to the common and ethical requirements for animal welfare. All parasitoids were carefully handled during experiments and maintained in the laboratory under appropriate conditions.

## Results

### Impact of yeasts on scent profiles

Analysis of the MVOCs that were collected from the different yeast-fermented nectars revealed differences in the nectar volatile composition and quantity (Table 1). In total, 13 MVOCs were detected for the different NIYs. As anticipated, the total amount of MVOCs emitted by the reference strain (Y182) was significantly higher compared to the other yeasts (*H*_6_ = 12.62, *P* < 0.001), particularly due to the high emission of acetaldehyde [*F*_(6, 13)_ = 272.67, *P* = 0.0036], 2-methyl propanol (*H*_6_ = 12.08, *P* = 0.0077) and 3-methyl-1-butanol [*F*_(6, 13)_ = 123.19, *P* = 0.0145]. PCA of the MVOCs showed that the first two components accounted for 77.8% of the total variation in volatile data (Figure 1A). Overall, PCA revealed that, compared to the control nectar, largest differences were for *A. pullulans, M. gruessii*, and *M. reukaufii*-fermented nectars. By contrast, the volatile blends emitted by *H. uvarum* and *S. roseus* only marginally differed from the control nectar. Furthermore, a noticeable separation was found between nectar fermented with NIYs and the reference strain Y182 (Figure 1A). The greatest loadings of PC1, in descending order, were for isoamyl acetate (0.335), ethyl propionate (0.334), isobutyl acetate (0.334) and 2-methyl propanol (0.329), whereas the greatest loadings of PC2 were for ethyl butyrate (0.425), 3-methyl-1-butanol (0.334), methanethiol (0.313) and 2-phenyl ethanol (0.253).

**Table 1:** Chemical profiles of synthetic nectar inoculated with various nectar-inhabiting yeasts.

**Category**	**Class**	**Compound**	**Unit**	Nectar-inhabiting yeasts^*^							***P*-value^#^**
				**Control**	**Y182**	**A.p**.	**H.u**.	**M.g**.	**M.r**.	**S.r**.	
Acidity			pH	5.76^a^	4.07^b^	3.91^c^	4.94^ab^	5.08^a^	4.48^b^	4.32^b^	**0.05**
Acids		Acetic acid	mg/l	0.004^b^	0.152^a^	0.008^b^	0.018^b^	0.023^b^	0.039^b^	0.047^b^	**0.0125**
		D-Lactic acid	mg/l	1.34^b^	11.41^a^	2.58^b^	1.46^b^	1.26^b^	1.23^b^	2.11^b^	**0.0250**
Amino acids	Acidic and their amides	Aspartic acid	mmol/l	234.73^b^	23.35^d^	280.42 ^a^	199.34^c^	214.27^b^	236.06^b^	262.7^a^	**0.0029**
		Asparagine	mmol/l	0.44^c^	1.96^a^	1.46^b^	0.61^c^	1.43^b^	1.27^b^	0.89^bc^	**0.0088**
		Glutamic acid	mmol/l	374.82^b^	15.14^c^	345.41^b^	345.45^b^	310.65^b^	310.34^b^	452.59^a^	**0.0059**
		Glutamine	mmol/l	0.26^c^	13.77^a^	7.12^b^	4.15^b^	1.89^bc^	2.85^bc^	4.86^b^	**0.0118**
	Aliphatic	Alanine	mmol/l	97.08^b^	153.11^a^	133.63^a^	116.6^ab^	65.92^c^	92.45^b^	130.07^a^	**0.0147**
		Arginine	mmol/l	36.94^a^	0.34^b^	17.31^a^	26.07^a^	35.14^a^	36.19^a^	18.9^a^	**0.0206**
		Glycine	mmol/l	60.93^a^	65.5^a^	64.15^a^	56.93^a^	48.09^b^	58.075^a^	69.87^a^	**0.0471**
		Iso-Leucine	mmol/l	60.11^ab^	62.72^ab^	64.21^ab^	79.46^a^	46.83^b^	52.69^b^	61.6^ab^	**0.0441**
		Leucine	mmol/l	102.22	94.98	112.45	119.74	81.93	91.32	113.59	0.05
		Valine	mmol/l	105.57^a^	149.02^a^	126.28^a^	149.36^a^	90.17^b^	103.67^a^	116.19^a^	**0.0353**
	Aromatic	Phenylalanine	mmol/l	53.62^b^	61.01^ab^	68.45^a^	61.56^ab^	40.71^c^	45.69^c^	63.23^a^	**0.0294**
		Tyrosine	mmol/l	30.41^a^	35.63^a^	36.42^a^	40.94^a^	26.16^b^	30.82^a^	35.71^a^	**0.0412**
	Basic	Histidine	mmol/l	24.07^a^	10.95^b^	22.31^a^	31.04^a^	22.79^a^	22.53^a^	25.9^a^	**0.0324**
		Lysine	mmol/l	106.91^a^	51.87^b^	64.67^b^	121.84^a^	107.28^a^	109.03^a^	120.38^a^	**0.0176**
	Containing OH group	Serine	mmol/l	118.35^a^	125.82^a^	95.16^b^	75.99^c^	65.41^c^	85.42^b^	110.58^ab^	**0.0235**
		Threonine	mmol/l	75.87^a^	75.03^a^	91.12^a^	80.85^a^	43.07^b^	54.49^b^	78.71^a^	**0.0265**
	Containing sulfur	Methionine	mmol/l	39.14^ab^	26.56^b^	27.67^b^	48.81^a^	26.46^b^	27.86^b^	34.34^b^	**0.0382**
MVOCs	Alcohol	2-Methyl propanol	mg/l	ND^c^	22.238^a^	3.537^b^	0.058^c^	0.038^c^	0.392^c^	0.036^c^	**0.0077**
		3-Methyl-1-butanol	mg/l	ND^d^	5.806^a^	1.172^bc^	0.167^d^	0.901^c^	1.777^b^	0.073^d^	**0.0145**
		2-Phenyl ethanol	mg/l	ND	1.461	0.318	0.377	1.643	0.391	0.203	0.0462
	Aldehyde	Acetaldehyde	mg/l	0.014^c^	21.987^a^	4.419^b^	0.262^c^	0.069^c^	0.467^c^	0.029^c^	**0.0038**
	Ester	Amyl acetate	mg/l	ND^b^	0.006^a^	ND^b^	ND^b^	0.006^a^	ND^b^	ND^b^	**0.0308**
		Ethyl acetate	mg/l	ND^c^	0.076^ab^	0.001^c^	0.096^a^	ND^c^	0.002^c^	0.035^b^	**0.0346**
		Isoamyl acetate	mg/l	ND^b^	0.084^a^	ND^b^	ND^b^	ND^b^	ND^b^	ND^b^	**0.0296**
		Propyl acetate	mg/l	ND^c^	0.035^a^	ND^c^	0.009^c^	ND^c^	ND^c^	0.018^b^	**0.0115**
		Isobutyl acetate	mg/l	ND^b^	0.022^a^	ND^b^	ND^b^	ND^b^	ND^b^	ND^b^	**0.0192**
		Ethyl butyrate	mg/l	ND^c^	0.048^b^	0.118^a^	ND^c^	ND^c^	0.011^c^	ND^c^	**0.0231**
		Ethyl propionate	mg/l	ND	0.038	ND	ND	ND	ND	ND	0.0324
	Containing sulphur	Dimethyl disulfide	μg/l	ND	1.037	ND	ND	ND	ND	0.019	0.0423
		Methanethiol	μg/l	ND	0.295	0.242	0.227	0.211	0.561	0.209	0.05
Sugars	Monosaccharide	Glucose	mmol/l	4.93^e^	208.68^b^	313.75^a^	25.87^d^	17.86^d^	19.01^d^	45.58^c^	**0.0167**
		Fructose	mmol/l	3.95^d^	224.57^a^	68.03^b^	26.68^c^	18.76^c^	21.17^c^	45.88^b^	**0.05**
	Disaccharide	Sucrose	mmol/l	434.01^b^	286.78^d^	45.81^e^	480.04^a^	403.11^c^	421.97^b^	419.57^b^	**0.0333**
Sulphur		Sulphur dioxide	mg/l	0.05^c^	0.29^a^	0.16^b^	0.07^c^	0.16^b^	0.12^bc^	0.23^ab^	**0.0375**

*Presented values are means of two biological replicates, on which two measurements were performed (two technical replicates). Different letters within rows indicate statistically significant differences (P ≤ 0.05); when no letters are present there were no significant differences between treatments*.

* *Nectar-inhibiting yeasts were: Control, non-inoculated, yeast-free nectar; Y182, fermented nectar with Saccharomyces cerevisiae; A.p., fermented nectar with Aureobasidium pullulans; H.u., fermented nectar with Hanseniaspora uvarum; M.g., fermented nectar with Metschnikowia gruessii; M.r., fermented nectar with M. reukauffii; S.r., fermented nectar with Sporobolomyces roseus. MVOCs were identified according to retention times on DB-WAX column in comparison with synthetic standards*.

# *Adjusted P-values as calculated after correcting for multiple comparisons by the Benjamini and Hochberg method; bold fonts indicate statistical significance*.

**Figure 1:**
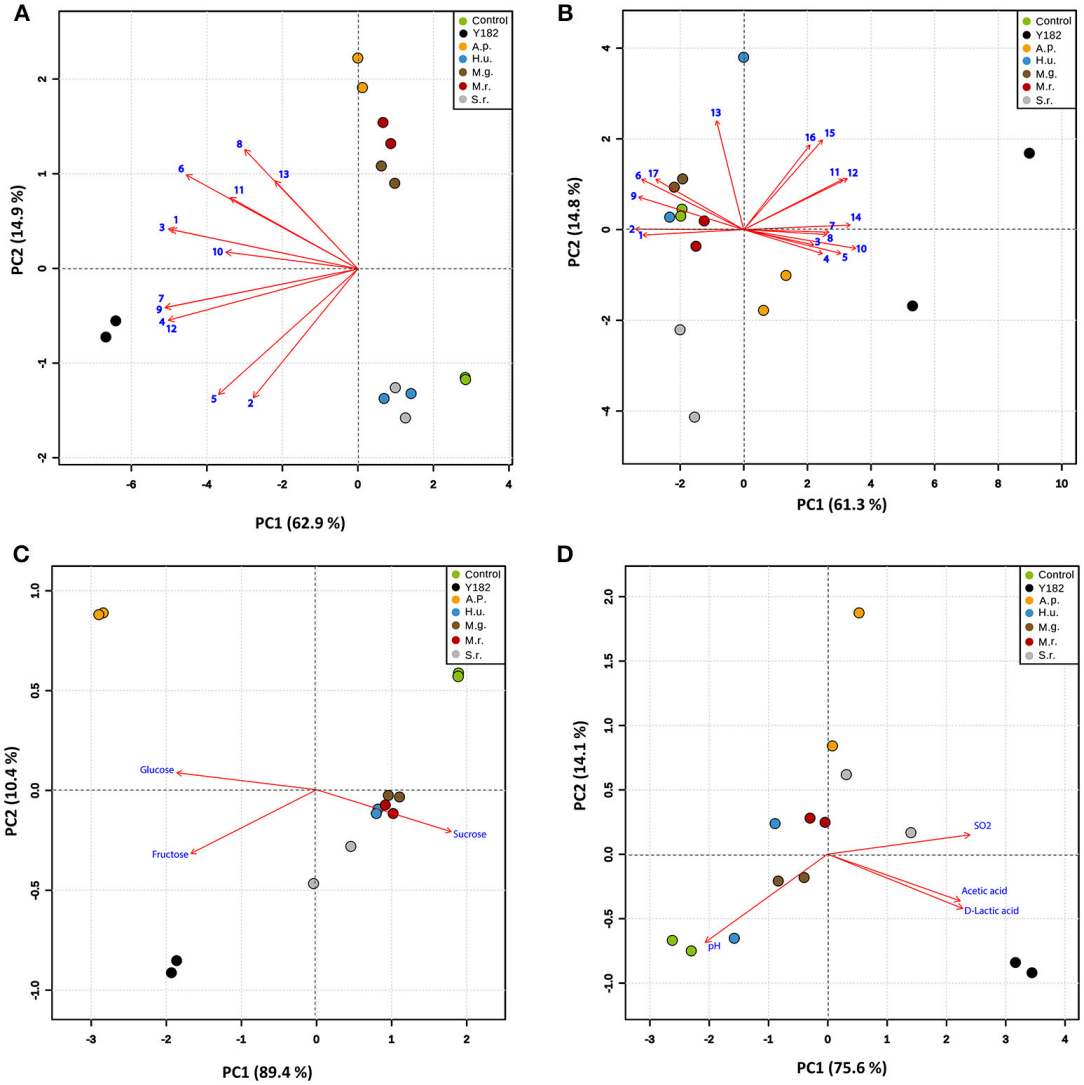
Principal component analysis (PCA) of the volatile and chemical profiles of the different nectars, including: Control, non-inoculated, yeast-free nectar; Y182, nectar fermented with the reference strain *Saccharomyces cerevisiae* Y182; A.p., *Aureobasidium pullulans*-fermented nectar; H.u., *Hanseniaspora uvarum*-fermented nectar; M.g., *Metschnikowia gruessii*-fermented nectar; M.r., *Metschnikowia reukaufii*-fermented nectar; S.r., *Sporobolomyces roseus*-fermented nectar. All analyses were performed on cell-free nectar solutions (two biological replicates, each with two technical replicates; mean values for each biological replicate are used in the analysis). Score plots visualize the location of each analyzed sample on each PC with the percentage of explained variation in parentheses, whereas vectors (in red) visualize the loadings for each variable. **(A)** PCA showing variation in microbial volatile composition across the different treatments. Vector numbers refer to the different volatile compounds: (1) Acetaldehyde, (2) Ethyl acetate, (3) 2-Methyl propanol, (4) Ethyl propionate, (5) Propyl acetate, (6) 3-Methyl-1-butanol, (7) Isobutyl acetate, (8) Ethyl butyrate, (9) Isoamyl acetate, (10) Amyl acetate, (11) 2-Phenyl ethanol, (12) Dimethyl disulfide, and (13) Methanethiol. **(B)** PCA showing variation in the amino acids composition across different treatments. Vector numbers refer to the different amino acids: (1) Aspartic acid, (2) Glutamic acid, (3) Asparagine, (4) Serine, (5) Glutamine, (6) Histidine, (7) Glycine, (8) Threonine, (9) Arginine, (10) Alanine, (11) Tyrosine, (12) Valine, (13) Methionine, (14) Phenylalanine, (15) Iso-Leucine, (16) Leucine and (17) Lysine. **(C)** PCA showing variation in the sugar composition across the different nectars investigated. **(D)** PCA showing variation in acidity (pH), and the acids and sulfur dioxide composition in the different nectars. The percentage of variation of the data explained by PC1 and PC2 is shown in parentheses (**A** volatiles: 62.9 & 14.9%; **B** Amino acids: 61.3 % & 41.8 %; **C** Sugars: 89.4 & 10.4 %; **D** Acids: 75.9 & 14.1 %, respectively).

### Impact of yeasts on nectar chemistry

Amino acids concentration and composition were significantly affected by inoculation of yeast strains (Table 1). In particular, Y182 and *M. gruessii* significantly reduced the total amino acids content [*F*_(6, 13)_ = 10.73, *P* = 0.003] by an average of 36.5 and 19.3%, respectively. In contrast, *S. roseus* was the only yeast that increased, albeit marginally, total amino acids content by an average of 11.7% compared to the control nectar, especially glutamic acid and alanine (Table 1). The multivariate analysis (PCA) of amino acids showed that the first two components accounted for 76.1% of the total variation in amino acids data (Figure 1B). Overall, PCA revealed a very clear separation among the amino acid profiles from Y182, *A. pullulans* and *S. roseus*-fermented nectar compared to the control and nectar fermented by the other tested yeasts (Figure 1B). The greatest loadings of PC1 were for alanine (0.297), phenylalanine (0.283) and valine (0.276) and, whereas the greatest loadings of PC2 were for methionine (0.543), iso-leucine (0.448) and leucine (0.422).

A similar trend was observed for sugars (Table 1). Both Y182 and *A. pullulans* significantly reduced sucrose (*H*_6_ = 11.943, *P* < 0.001) concentrations by an average of 33.9 and 89.5%, respectively, compared to the other nectars including the control. Furthermore, all yeast strains significantly increased glucose and fructose concentrations, especially Y182 and *A. pullulans* (Table 1). PCA showed that the first two components accounted for 99.8% of the total variation in the sugars data. Again, the largest separation was seen for Y182 and *A. pullulans* where both fructose and glucose vectors were more associated with the samples of these yeast-fermented nectars (Figure 1C), whereas the sucrose vector was more associated with the control and other tested yeasts.

Furthermore, it was found that all tested yeast strains significantly decreased nectar pH [*F*_(6, 13)_ = 10.74, *P* = 0.05], particularly Y182 and *A. pullulans* which reduced the pH from 5.76 to 4.07 and 3.91, respectively (Table 1). Additionally, Y182 drastically increased the concentration of acetic acid [*F*_(6, 13)_ = 28.92, *P* = 0.0125], D-Lactic acid [*F*_(6, 13)_ = 28.42, *P* = 0.0250] and sulfur dioxide [*F*_(6, 13)_ = 16.26, *P* = 0.0375] compared to the other tested yeasts. The multivariate analysis (PCA) of these compounds showed that the first two components accounted for 89.7% of the total variation in organic acids and sulfur dioxide data (Figure 1D), and clearly demonstrated differences between the control nectar and nectar fermented by yeasts, especially for Y182 and *A. pullulans*.

### Impact of yeast-fermented nectars on insect behavioral response

**Figure 2:**
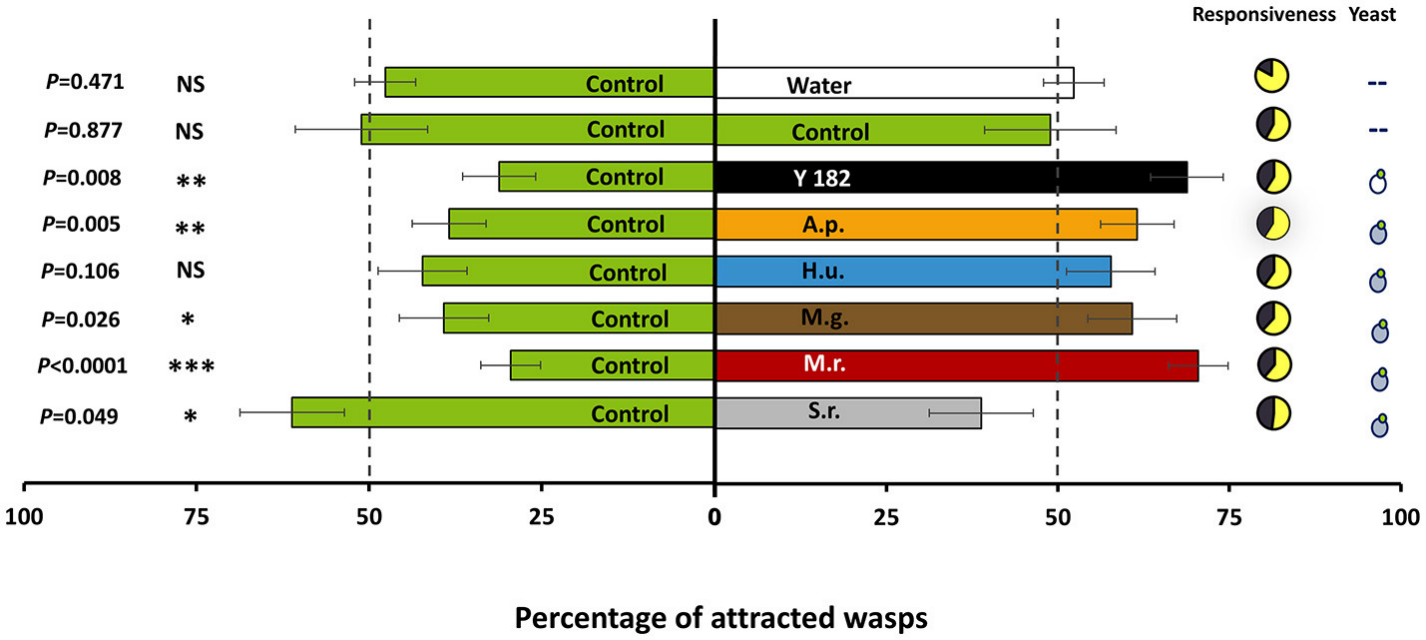
Olfactory response of adult *Aphidius ervi* females when given the choice between two odors (percentage ± SE, *n* = 100). Treatments included: Control, non-inoculated, yeast-free nectar; Water, distilled water; Y182, nectar fermented with the reference strain *Saccharomyces cerevisiae* Y182; A.p., *Aureobasidium pullulans*-fermented nectar; H.u., *Hanseniaspora uvarum*-fermented nectar; M.g., *Metschnikowia gruessii*-fermented nectar; M.r., *Metschnikowia reukaufii*-fermented nectar; S.r., *Sporobolomyces roseus*-fermented nectar. Nectar-inhabiting yeasts are marked with a blue yeast-like symbol, whereas the reference strain is marked with a white yeast-like symbol. Experiments were performed with cell-free nectars. The bioassay was carried out by releasing 20 groups of five females at the base of a two-choice Y-olfactometer and evaluating their response 10 min after their release. Wasps that passed a set line at the end of the olfactometer arms and were still there at the time of evaluation were considered to have chosen for the odor source connected to that olfactometer arm. Parasitoids that did not make a choice within 10 min after release were considered as non-responding individuals, and were excluded from the statistical analysis. Pie charts show the distribution of responding (in yellow) and non-responding (in black) individuals. Asterisks indicate a preference that is significantly different (*t*-test) from a 50:50 distribution within a choice test: ^***^P < 0.001; ^**^0.001 ≤ *P* < 0.01; ^*^0.01 ≤ *P* ≤ 0.05; NS, non-significant.

Overall, yeast-fermented nectar elicited strong attraction of *A. ervi* parasitoid females compared to the control in a binary choice assay [*t*_(38)_ = 2.240, *P* = 0.026; Figure 2]. Of the six yeast strains tested, four strains showed significant enhanced attraction of *A. ervi*, among which *M. reukaufii* evoked the most significant response [*t*_(38)_ = 6.512, *P* < 0.001], followed by the reference strain Y182 [*t*_(38)_ = 2.800, *P* = 0.008], *A. pullulans* [*t*_(38)_ = 2.976, *P* = 0.005], and *M. gruessii* [*t*_(38)_ = 2.303, *P* = 0.027]. By contrast, parasitoid females showed a significant negative response to *S. roseus* [*t*_(38)_ = 2.029, *P* = 0.047], indicating a repellent effect. In addition, no attraction or repellency was recorded for parasitoid females toward *H. uvarum* [*t*_(38)_ = −1.656, *P* = 0.106]. The equal distribution of parasitoids when both odor sources were provided with the control nectar demonstrated that there was no positional bias within our experimental set-up [*t*_(38)_ = 0.156, *P* = 0.877]. Additionally, parasitoid females showed similar response to the control treatment or to water [*t*_(38)_ = 0.727, *P* = 0.472; Figure 2], indicating that the nectar medium itself has no repellent or attractant effect on the parasitoids.

### Impact of yeast-fermented nectars on nectar intake, longevity and survival

**Figure 3:**
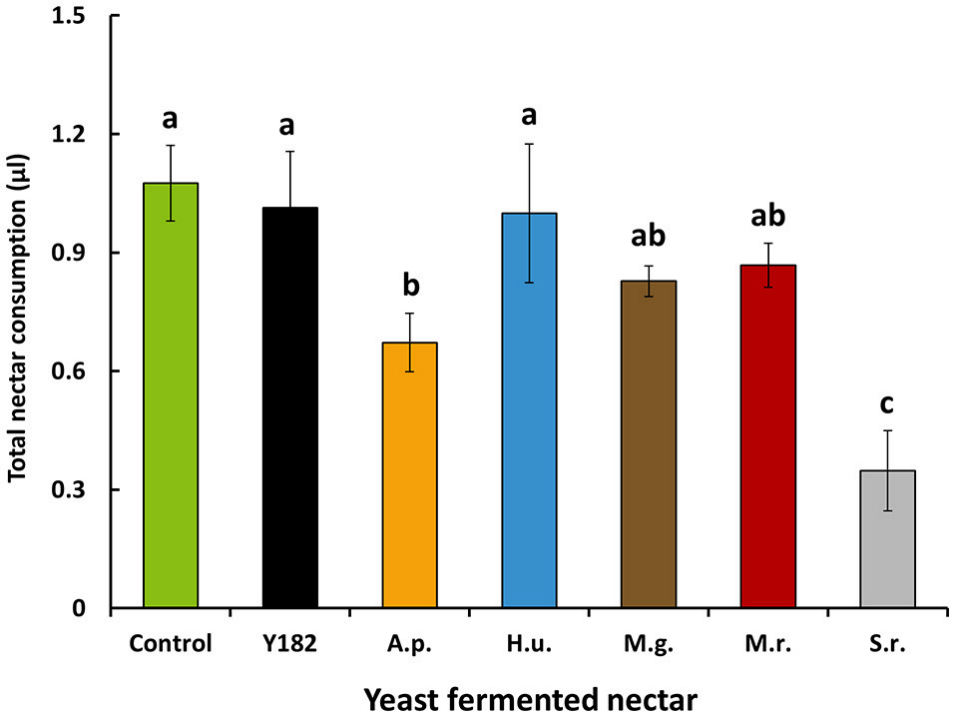
Mean nectar intake (± SE, *n* = 5, each with 15 individuals per cage) by feeding-inexperienced adult *Aphidius ervi* parasitoids after 9 h of nectar supply. Parasitoids were provided different nectars, including: Control, non-inoculated, yeast-free nectar; Y182, nectar fermented with the reference strain *Saccharomyces cerevisiae* Y182; A.p., *Aureobasidium pullulans*-fermented nectar; H.u., *Hanseniaspora uvarum*-fermented nectar; M.g., *Metschnikowia gruessii*-fermented nectar; M.r., *Metschnikowia reukaufii*-fermented nectar; S.r., *Sporobolomyces roseus*-fermented nectar. Experiments were performed using cell-free nectar solutions. Different letters above colored bars indicate significant differences between provided nectars (*P* < 0.05), based on Student-Newman-Keuls method (*F*-test).

**Figure 4:**
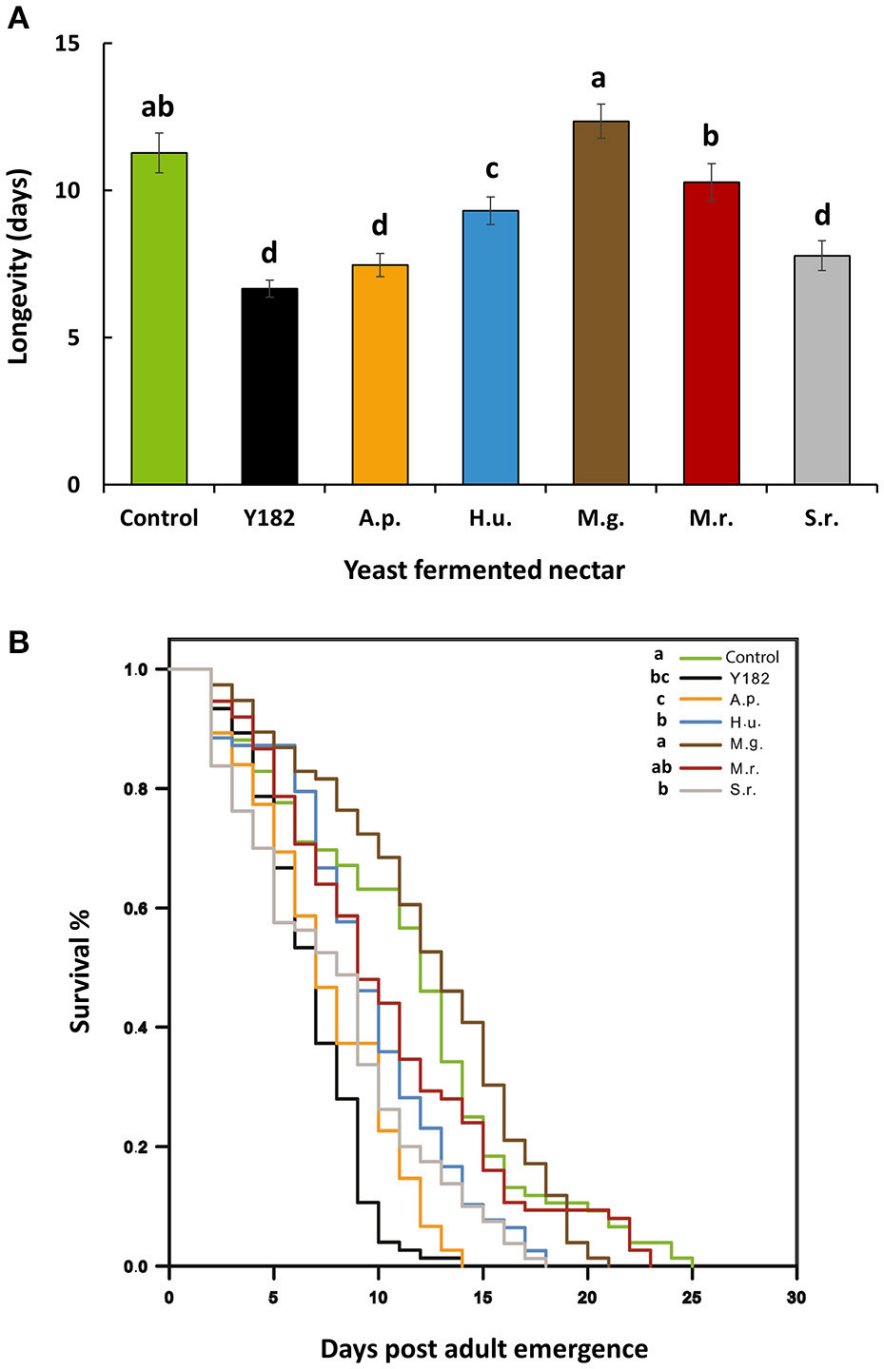
**(A)** Mean longevity (± SE, *n* = 75, equally distributed over 5 containers) and **(B)** Kaplan–Meier survival curves of *Aphidius ervi* adults fed on different nectars, including: Control, non-inoculated, yeast free nectar; Y182, nectar fermented with the reference strain *Saccharomyces cerevisiae* Y182; A.p., *Aureobasidium pullulans*-fermented nectar; H.u., *Hanseniaspora uvarum*-fermented nectar; M.g., *Metschnikowia gruessii*-fermented nectar; M.r., *Metschnikowia reukaufii*-fermented nectar; S.r., *Sporobolomyces roseus*-fermented nectar. Experiments were performed with cell-free nectar samples under laboratory conditions of 22°C, 70% relative humidity and a 16:8 h light:dark photoperiod. Capillaries with nectar solutions were replaced daily to avoid microbial contamination. Different letters on bars in **(A)** indicate significant differences between provided nectars (*P* < 0.05), based on Student-Newman-Keuls method (*F*-test). In **(B)** different letters indicate significant differences between curves (pairwise log–rank *post-hoc* tests with Bonferroni correction, *P* < 0.05, *n* = 75).

Total nectar consumption (the total amount of nectar consumed measured over a total period of 9 h) significantly differed between nectars [*F*_(6, 28)_ = 5.52, *P* < 0.001]. More specifically, intake of *S. roseus*-fermented nectar was 3-fold less than the control nectar, which was also the case, but to lesser extent, for *A. pullulans*-fermented nectar (Figure 3). Similar to nectar intake, yeast-fermented nectars had a significant impact on parasitoid life span [*F*_(6, 28)_ = 16.19; *P* < 0.001; Figure 4A] and survival (Log-rank test = 112.54, *df* = 6; *P* < 0.001; Figure 4B). Specifically, yeast inoculation significantly reduced parasitoid longevity with 6.6, 7.4, 7.7, and 9.3 days when parasitoids were fed on nectar fermented with Y182, *A. pullulans, S. roseus* and *H. uvarum*, respectively (Figure 4A). In contrast, no differences in longevity were observed compared to the control when parasitoids were fed on nectar fermented with the nectar specialists *M. gruessii* and *M. reukaufii* (Figure 4A).

## Discussion

Here, we demonstrate that specialist, but not generalist, nectar inhabiting yeasts that rely on flower foraging insects for their dispersal produce attractive scent profiles for a generalist aphid parasitoid without affecting its survival and longevity.

### Impact on scent profiles and behavioral response

Our results clearly show that NIYs significantly change the scent profile of nectar and that there was considerable variation between yeast species, suggesting that NIYs emit volatile blends that are to a large extent species specific, corroborating earlier findings (Rering et al., 2017). Volatiles produced by NIYs are mainly byproducts or secondary metabolites of the yeast metabolism or fermentation but may have diverse ecological functions (Dzialo et al., 2017). For example, volatile compounds such as ethyl acetate, 2-butanol, isobutanol, ethanol, 2-ethyl-1-hexanol and 2-phenylethanol have been shown to inhibit microbial growth (Cruz et al., 2012; Hua et al., 2014; Pereira et al., 2016), and may help explain why earlier nectar-colonizers often suppress the growth of later arriving microbial species (Peay et al., 2012; Vannette and Fukami, 2014). Furthermore, microbes that rely on insects for dispersal or survival may produce volatiles that are attractive to the insect vectors (Dzialo et al., 2017). For example, compounds like 3-methyl-1-butanol and 2-phenyl ethanol, which are commonly produced by many yeasts including those investigated in this study, are very attractive to a wide diversity of insects (Davis et al., 2013), including hymenopteran insects (Davis et al., 2012; Rering et al., 2017).

Yeasts like *M. gruessii* and *M. reukaufii* are highly abundant nectar specialists (Pozo et al., 2011) that largely rely on floral visitors for dispersal among flowers (Brysch-Herzberg, 2004; Belisle et al., 2012). Furthermore, it was recently found that these specialist yeasts rely on multiple floral visits and repeated inoculations in the nectar to establish their dominant abundance in the nectar microbial community (Mittelbach et al., 2016). Therefore, it is reasonable to expect that these yeasts produce attractive volatiles that aid in their dispersal. Indeed, bumblebees not only responded positively to flowers colonized by *M. reukaufii* (Schaeffer et al., 2014), but also spent significantly longer foraging time on *M. reukaufii*-inoculated flowers compared to yeast-free flowers (Schaeffer et al., 2017). Interestingly, this robust attraction to *Metschnikowia* spp. has also been reported for pest insects (Witzgall et al., 2012). Additionally, *M. reukaufii* has been shown to produce a distinct volatile blend which was the most attractive to honey bees among different microorganisms tested (Rering et al., 2017). In line with these observations, we also found that parasitoid females were attracted the most to *M. reukaufii-*fermented nectar, followed by the reference *S. cerevisiae* strain Y182, *A. pullulans*, and *M. gruessii*. In contrast to *M. gruessii* and *M. reukaufii*, the other tested yeasts (i.e., *A. pullulans, H. uvarum* and *S. roseus*) are ubiquitous yeasts that are associated with a wide diversity of habitats, including diverse aerial plant parts (Andrews et al., 1994; Nakase, 2000; Jolly et al., 2014). It can therefore be hypothesized that these yeasts are less dependent on insect vectors or differ in dispersal vectors, and therefore produce different or lower amounts of volatile compounds. Except for the results with *A. pullulans*, which also showed a strong parasitoid attraction, our results support this hypothesis. Indeed, in contrast to the other yeasts tested, both *H. uvarum* and *S. roseus* did not produce high levels of volatiles, and were also not attractive to *Aphidius* parasitoids. *S. roseus* was even found to be deterrent to *A. ervi*. In line with our results, *A. pullulans* has also been previously reported to produce volatile compounds that attract insects (Davis and Landolt, 2013; Hung et al., 2015). The PCA of scent profiles provided further indications on which compounds may be of importance for parasitoid attraction. In particular, isoamyl acetate, isobutyl acetate, 2-methyl propanol, 3-methyl-1-butanol and 2-phenyl ethanol had the greatest loadings for PC1 and PC2, suggesting that production of these compounds correlates most strongly with parasitoid attraction. However, further research with pure chemical compounds is needed to unravel their exact contribution to parasitoid attraction. Moreover, it is not unreasonable to assume that the insect's behavior will depend on blends of these MVOCs, rather than on a single compound, as has been shown for plant volatiles (Takemoto and Takabayashi, 2015).

In addition to volatile compounds that may have an effect on insect behavior, amino acids may also have a notable effect on insect chemoreceptors (Hansen et al., 1998; Carter et al., 2006). In particular, it was found that glutamic acid, leucine and methionine have the potential to modify insect behavior by stimulating insect chemosensory orientation (Wacht et al., 2000). Strikingly, yeast-fermented nectars that showed a non-attractive or repellent response to *A. ervi* females (i.e., *H. uvarum* and *S. roseus*) produced these amino acids in high concentrations, suggesting a potential role for these amino acids in the parasitoids rejection of these nectars. Interestingly, further supporting explanation is provided by the multivariate analysis which disclosed that glutamic acid, leucine and methionine were among the greatest loadings in the first two PCs, highlighting their potential contribution to shape the parasitoid behavior.

### Impact on nectar chemistry, nectar intake, and survival

Overall, our results show that NIYs strongly affect nectar sugar and amino acids composition and concentration, thereby corroborating previous findings. Interestingly, whereas the different yeast strains depleted several amino acids (arginine, aspartic acid, histidine, serine and threonine) compared to the control nectar, increased concentrations of specific amino acids such as asparagine, alanine, glutamine and methionine were also detected. Moreover, NIYs considerably impacted nectar acidity with a manifest drop in pH from 5.76 to even 3.91 following the inoculation of *A. pullulans*. As a result, it can be expected that such changes in nectar chemistry may impact the overall nectar's appeal and nutritional value (Petanidou, 2005; Nicolson and Thronburg, 2007; Gijbels et al., 2014), thereby potentially also affecting life history parameters such as longevity (Lenaerts et al., 2017). When parasitoids were provided the various yeast-fermented nectars, their nectar intake was distinctly affected. While none of the tested nectars showed enhanced consumption relative to the control nectar, nectars fermented with *S. roseus* and *A. pullulans* were consumed significantly less. One potential explanation for this reduced consumption could be the change in amino acid profile caused by these two yeasts compared to the control (Hendriksma et al., 2014). Overall, *S. roseus* was the only yeast that increased the total amino acid content (Table 1). Further, both *S. roseus* and *A. pullulans* increased the amount of aspartic acid and phenylalanine compared to the control. Recently, it has been shown that relatively high concentrations of amino acids such as phenylalanine may inhibit feeding on sucrose solutions containing them, and can act as inhibitors during associative learning (Simcock et al., 2014). This may provide a potential explanation for the reduced consumption of nectar fermented by *S. roseus* and *A. pullulans*. In addition, inoculation with these yeasts resulted in a reduction of pH and a distinct acidity profile. It has been shown that many pollinators avoid acidic nectars (Vannette et al., 2013; Good et al., 2014; Junker et al., 2014). By contrast, other insects such as fruit flies seem to prefer an acidic diet over neutral or alkaline pH food (Deshpande et al., 2015).

In line with nectar intake, NIYs also significantly affected the survival and longevity of the parasitoids. Particularly, parasitoids that fed on *S. roseus, A. pullulans* and Y182-fermented nectars showed shortest longevity and lowest survival, suggesting that these nectars lack important nutrients or contain one or more unsuitable compounds. Interestingly, these yeasts consumed more sucrose and simultaneously produced higher amounts of glucose and fructose compared to the other yeasts. Likewise, reduced longevity was observed for *A. ervi* adults fed on nectar inoculated with the bacterium *Asaia* sp. which similarly decreased the sucrose concentration whereas the glucose and fructose content increased (Lenaerts et al., 2017). Similarly, bees and eusocial wasps preferred nectars that contain a high amount of sucrose (Petanidou, 2005). Although sucrose and its hexose components glucose and fructose are considered very suitable carbohydrate sources for most hymenopteran parasitoids (Wäckers, 2001; Luo et al., 2010), further research is needed to find out whether the absolute content of sucrose, glucose and fructose affects nectar consumption and survival of *Aphidius* wasps. By contrast, parasitoid longevity and survival was not affected by inoculation of both *Metschnikowia* species. Similarly, a recent study has shown that *M. reukauffii* had no adverse effects on bumble bee reproduction, including initiation of egg laying and number of eggs laid (Schaeffer et al., 2017).

It has to be noted that we only examined effects of the chemical changes induced by the NIYs by testing cell-free nectar media, while direct effects of the microbes were not considered. It is generally accepted that the microbes themselves can also provide insects with many benefits, e.g., acting as a nutrition source, detoxifying harmful substances, protection from biotic stresses (Crotti et al., 2009; Gibson and Hunter, 2010; Vannette and Fukami, 2016). Further, potential plant effects were not taken into account. In this regard, it may be possible that the effects observed in this study may be different from those seen in field studies or in-flower inoculations, as plants may also influence nectar chemistry (Canto et al., 2017; Vannette and Fukami, 2018).

### Potential applications

Recently, there is an increasing interest in harnessing insect-microbe chemical communications to control insect pests in agricultural systems (Davis et al., 2013; Beck and Vannette, 2017). In particular, it has been shown that MVOCs produced by yeasts robustly mediate host finding and food location for a wide range of insects (Dzialo et al., 2017), including sap beetles (Nout and Bartelt, 1998), codling moth (Witzgall et al., 2012), spotted wing drosophila (Scheidler et al., 2015; Mori et al., 2017), European grapevine moth (Tasin et al., 2011) and coffee bean weevil (Yang et al., 2017). Interestingly, these findings promote the possibility of exploiting yeast-based attraction as an ecofriendly technique to control pest insects, e.g., by luring them away from the crop or attract and kill them using specific traps (Davis and Landolt, 2013; Andreadis et al., 2015). Based on our results, a similar strategy could be developed to attract natural enemies into the field and prevent pest populations from reaching the economic injury level. In this regard, further study should focus on the specificity of the interactions to ensure only beneficial insects are attracted.

## Conclusions

Overall, our results indicate that nectar yeasts modulate floral nectar attractiveness to flower-visiting insects by producing distinctive scent profiles. Furthermore, we have demonstrated that feeding on these fermented nectars affected insect longevity and survival. Interestingly, our results support the hypothesis that microorganisms that almost solely occur in nectar and that are therefore strongly dependent on floral visitors for dispersal produced volatile compounds that enhance insect attraction. Additionally, we showed that these microorganisms had no adverse effects on the longevity and survival of their vectors. Nevertheless, the exact consequences of altered insect behavior for the yeasts, the insects, and also the plants, still remain unclear to date and requires further study. Additionally, we only examined responses of the generalist parasitoid *A. ervi*, so it is possible that other flower-visiting insects respond differently. Our results also provide support to recent suggestions that secondary metabolites signaling between yeasts and insects can be used as a promising tool for sustainable crop protection, e.g., to improve methods currently used in controlling or monitoring insect pests (Beck and Vannette, 2017). Further research is needed to investigate the feasibility of such strategy.

## Author contributions

ISS, HJ, and BL conceived the ideas and designed the experiments. ISS, DB, TG, BH-M, and LB performed the experiments and collected the data. ISS, DB, HJ, and BL analyzed the data. FW and KV contributed to equipment, reagents and materials. BH-M and WV contributed to nectar chemical analysis. ISS, HJ, and BL led the writing of the manuscript. All authors contributed critically to the drafts and gave final approval for publication. The authors have declared that no competing interests exist.

### Conflict of interest statement

The authors declare that the research was conducted in the absence of any commercial or financial relationships that could be construed as a potential conflict of interest. The reviewer FAS and handling Editor declared their shared affiliation.
