# Supplementary material for: Sweet Scents: Nectar Specialist Yeasts Enhance Nectar Attraction of a Generalist Aphid Parasitoid Without Affecting Survival
Source: Front Plant Sci. 2018 Jul 16;9:1009. doi: 10.3389/fpls.2018.01009 (PMC6055026; doi:10.3389/fpls.2018.01009)
Supplement: Supplementary file 2 [file Presentation_1.pptx]

## Slide 1
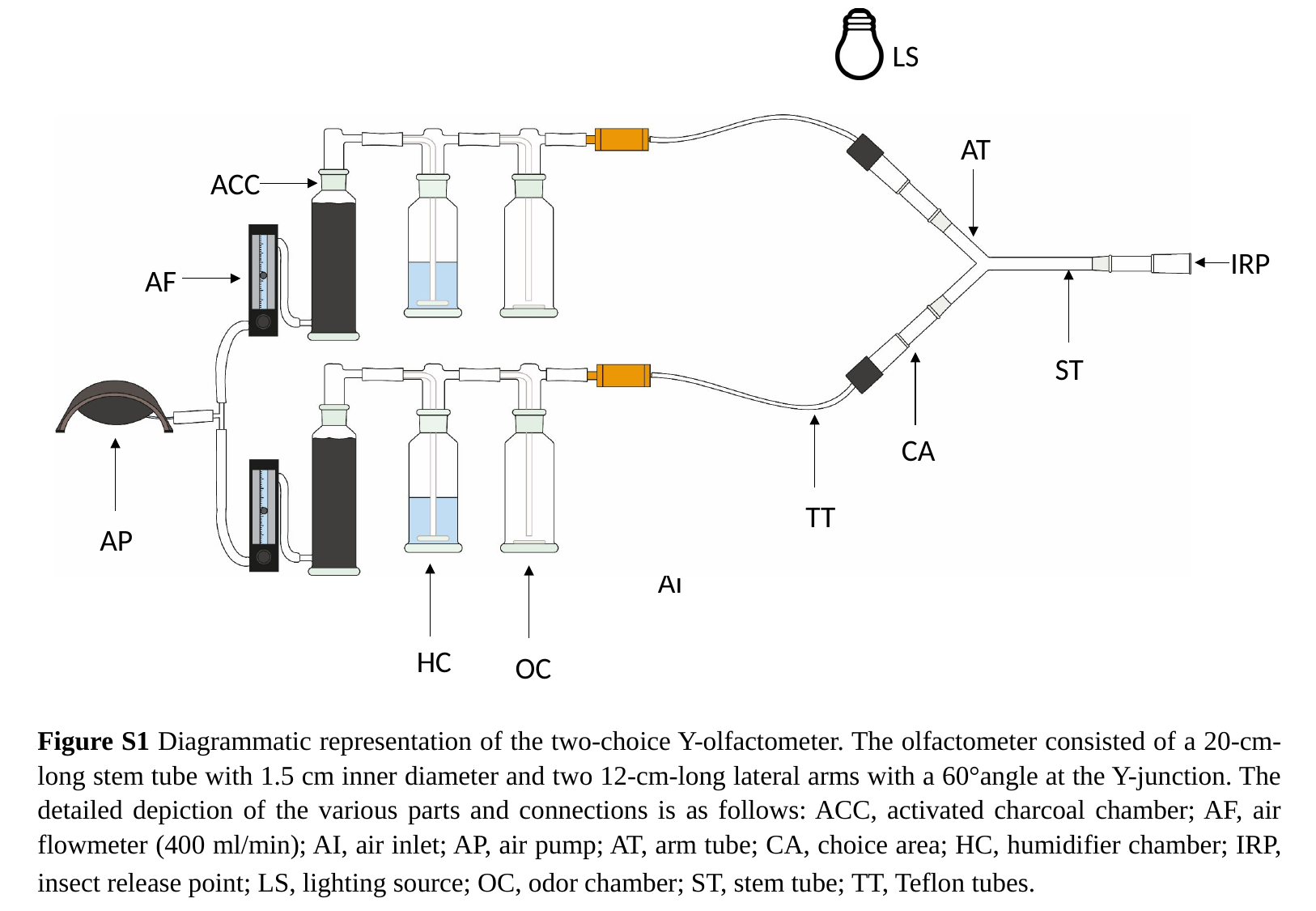

LS
AT
ACC
IRP
AF
ST
CA
TT
AP
HC
OC
Ai
Figure S1 Diagrammatic representation of the two-choice Y-olfactometer. The olfactometer consisted of a 20-cm-long stem tube with 1.5 cm inner diameter and two 12-cm-long lateral arms with a 60°angle at the Y-junction. The detailed depiction of the various parts and connections is as follows: ACC, activated charcoal chamber; AF, air flowmeter (400 ml/min); AI, air inlet; AP, air pump; AT, arm tube; CA, choice area; HC, humidifier chamber; IRP, insect release point; LS, lighting source; OC, odor chamber; ST, stem tube; TT, Teflon tubes.
