# Supplementary material for: Sweet Scents: Nectar Specialist Yeasts Enhance Nectar Attraction of a Generalist Aphid Parasitoid Without Affecting Survival
Source: Front Plant Sci. 2018 Jul 16;9:1009. doi: 10.3389/fpls.2018.01009 (PMC6055026; doi:10.3389/fpls.2018.01009)
Supplement: Supplementary file 3 [file Table_1.docx]

**Table S1. Nectar-inhabiting yeast strains used in this study.**

| **Strain  (GenBank Accession N°)** | **Phylogenetic affiliation^a^** | | | | | | **Origin (nectar)** | **Geographic origin (year of isolation)** | **Reference** |
| --- | --- | --- | --- | --- | --- | --- | --- | --- | --- |
|  | **Phylum** | **Family** | **Closest match in GenBank (Accession N°)^b^** | **Sequence identity (%)** | **E-value** | **Score** |  |  |  |
| ST12.14/075  (KP405904) | Basidiomycota | Sporobolomycetaceae | *Sporobolomyces roseus* (MF927664.1) | 504/504 (100.0) | 0.0 | 931 | *Epipactis helleborine* | Mirwart, Luxemburg, Belgium (2011) | (Jacquemyn et al., 2013) |
| EHE_1_Y1  (KC407605) | Basidiomycota | Saccharomycetaceae | *Hanseniaspora uvarum* (MG017583.1) | 497/497 (100.0) | 0.0 | 918 | *Centaurea cyanus* | Pulderbos, Antwerp, Belgium (2013) | (Lenaerts et al., 2016) |
| ST12.14/048  (KP405909) | [Ascomycota](https://en.wikipedia.org/wiki/Ascomycota) | Aureobasidiaceae | *Aureobasidium pullulans* (NG_055734.1) | 531/532 (99.8) | 0.0 | 977 | *Centaurea cyanus* | Pulderbos, Antwerp, Belgium (2013) | (Lenaerts et al., 2016) |
| ST12.14/016  (MG725249) | [Ascomycota](https://en.wikipedia.org/wiki/Ascomycota) | *Metschnikowiaceae* | *Metschnikowia gruessii* (KY108474.1) | 514/514 (100.0) | 0.0 | 950 | *Symphytum officinale* | Pulderbos, Antwerp, Belgium (2013) | (Sobhy et al., 2018) |
| ST12.14/017  (MG725250) | [Ascomycota](https://en.wikipedia.org/wiki/Ascomycota) | *Metschnikowiaceae* | *Metschnikowia reukaufii* (JX067775.1) | 441/441 (100.0) | 0.0 | 811 | *Symphytum officinale* | Pulderbos, Antwerp, Belgium (2013) | (Sobhy et al., 2018) |

^a^ Based on BLAST analysis of partial large subunit (LSU) ribosomal RNA gene sequences.

Jacquemyn, H., Lenaerts, M., Tyteca, D., and Lievens, B. (2013). Microbial diversity in the floral nectar of seven Epipactis (Orchidaceae) species. *Microbiologyopen* 2, 644–658. doi:10.1002/mbo3.103.

Lenaerts, M., Pozo, M. I., Wackers, F., van den Ende, W., Jacquemyn, H., and Lievens, B. (2016). Impact of microbial communities on floral nectar chemistry: Potential implications for biological control of pest insects. *Basic Appl. Ecol.* 17, 189–198. doi:10.1016/j.baae.2015.10.001.

Sobhy, I. S., Baets, D., Goelen, T., Herrera-malaver, B., Bosmans, L., Van den Ende, W., et al. (2018). Sweet Scents: Nectar Specialist Yeasts Enhance Nectar Attraction of a Generalist Aphid ParasitoidWithout Affecting Survival. *Front. Plant Sci.* 9, 1009. doi:10.3389/fpls.2018.01009.
